# Influence of Glyphosate Formulations on the Behavior of Sulfentrazone in Soil in Mixed Applications

**DOI:** 10.3390/toxics8040123

**Published:** 2020-12-17

**Authors:** Ana Cláudia Langaro, Matheus de Freitas Souza, Gustavo Antônio Mendes Pereira, João Pedro Ambrósio Barros, Antonio Alberto da Silva, Daniel Valadão Silva, Ana Beatriz Rocha de Jesus Passos, Vander Mendonça

**Affiliations:** 1 Instituto de Agronomia, Universidade Federal Rural do Rio de Janeiro, Seropédica, Rio de Janeiro 23897-000, Brazil; namelia.langaro@gmail.com; 2 Departamento de Ciências Agronômicas e Florestais, Universidade Federal Rural do Semi-Árido, Mossoró, Rio Grande do Norte 59625-900, Brazil; freitasouza@yahoo.com.br (M.d.F.S.); daniel.valadao@ufersa.edu.br (D.V.S.); vander@ufersa.edu.br (V.M.); 3 Departamento de Agronomia, Universidade Federal de Viçosa, Viçosa, Minas Gerais 36570-900, Brazil; gustavogamp@hotmail.com (G.A.M.P.); joao.ambrosio@ufv.br (J.P.A.B.); aasilva@ufv.br (A.A.d.S.)

**Keywords:** environmental impact, leaching, pesticide dynamics, soil contamination, sorption

## Abstract

The selection of weed biotypes that are resistant to glyphosate has increased the demand for its use mixed with other herbicides, such as sulfentrazone. However, when chemical molecules are mixed, interactions may occur, modifying the behavior of these molecules in the environment, such as the sorption and desorption in soil. In this study, we hypothesized that the presence of glyphosate-formulated products might increase the sorption or decrease the desorption of sulfentrazone, thereby increasing the risk of the contamination of water resources. Therefore, our work aimed to evaluate the sorption, desorption, and leaching of sulfentrazone in the soil in an isolated and mixed application with different glyphosate formulations. The sorption coefficients (Kfs) for the sulfentrazone, sulfentrazone + Roundup Ready, sulfentrazone + Roundup Ultra, and sulfentrazone + Zapp Qi foram were 1.3, 2.1, 2.3, and 1.9, respectively. The desorption coefficients (Kfd) for the sulfentrazone, sulfentrazone + Roundup Ready, sulfentrazone + Roundup Ultra, and sulfentrazone + Zapp Qi foram were 65.7, 125.2, 733.3 and 239.8, respectively. The experiments demonstrated that the sorption and desorption of sulfentrazone in combination with the other formulated glyphosate products are altered, supporting the hypothesis suggested by this work, i.e., that the presence of other molecules is a factor that affects the behavior of herbicides in the soil. This phenomenon altered the vertical mobility of sulfentrazone. Situations involving mixtures of pesticides should be evaluated in order to improve our understanding of the dynamics of these molecules and thus avoid environmental contamination.

## 1. Introduction

Weed management is an essential practice in production systems because it determines the yield productivity and quality of agricultural products. Among the control methods, chemical control is the most commonly-used in weed management due to its high efficiency, low cost, and practicality. Regardless of the application mode, pre- or post-emergence of weeds, the final destination of an herbicide is usually the soil [1]. In the soil, herbicides can undergo several processes (retention, transport, and transformation). They are influenced by the physical and chemical properties of the herbicide and the soil, and the climatic conditions of the region [2]. The retention (sorption and desorption) of an herbicide to soil colloids is a factor that defines the intensity of other processes. For example, the high sorption and low desorption of an herbicide reduces its mobility. On the other hand, soils with low sorption and high desorption present a more significant potential for the leaching and surface runoff of herbicides, contaminating water resources [3,4,5,6]. Therefore, knowledge of the interactions between the soil and a herbicide is critical to the reduction of the environmental impacts caused by the agrochemical’s applications.

Several studies have already shown how changes in the physical and chemical properties of soil can affect the behavior of herbicides in the soil [7,8,9]. Although many studies have evaluated the behavior of herbicides in soils with different physicochemical properties, these studies consider only the isolated presence of the herbicide, disregarding the fact that—in many situations—these agrochemicals are applied as a mixture with other pesticides. A survey among farmers in Brazil has shown that, 97% of the time, tank mixing is performed in the field [10]. Furthermore, Gazziero (2015) [10] reported that glyphosate applications to soybean are frequently (86%) carried out simultaneously with insecticides, fungicides and other herbicides.

The combined application of different herbicides has been driven by an increase in populations that are resistant and tolerant to glyphosate. This is an important practice for the integrated management of these plants. Among herbicides mixed with glyphosate, sulfentrazone [N-(2,4-dichloro-5-(4-(difluoromethyl)-4,5-dihydro-3-methyl-5-oxo-1H-1,2,4-triazol-1-yl)phenyl)methanesulfonamide] is an inhibitor of the enzyme protoporphyrinogen oxidase, and is capable of controlling resistant and tolerant species such as *Bidens pilosa*, *Euphorbia heterophylla*, *Ipomea grandifolia* and *Commelina benghalensis* [11,12]. Therefore, in areas with the presence of these species, sulfentrazone is commonly mixed with glyphosate at the time of desiccation in order to obtain the pre-emergence control of the species that are not controlled by glyphosate, allowing the producer to increase the control spectrum and reduce operating costs [13].

Although the combined application of herbicides is a common practice in agricultural fields, the fact has not been considered that this application modality can alter the behavior of these molecules in the soil. However, safety recommendations should consider all of the possible interactions between the components. It is necessary to know the interactions between the active ingredients and other compounds (adjuvants, surfactants, and inerts) that make up the formulated product, and whether they may also affect the herbicide’s behavior in the soil.

In this study, we hypothesized that the presence of glyphosate-formulated products may increase the sorption or decrease the desorption of sulfentrazone, increasing the risk of the contamination of water resources. Therefore, the objective of our work is to evaluate the sorption, desorption, and leaching of sulfentrazone in soil in isolated applications and mixed with different glyphosate formulations. We hope to demonstrate some interactions between herbicides that were previously not considered, and we believe that the results obtained could provide relevant information for the safety recommendations of these agrochemicals, reducing the environmental impact caused by their application.

## 2. Material and Methods

Two experiments were carried out, one in a laboratory and another in a greenhouse, in order to evaluate the behavior of sulfentrazone in isolated applications and mixed with glyphosate formulations. The first experiment was carried out in the laboratory in order to determine the sorption and desorption of sulfentrazone. The second experiment was conducted in a greenhouse in order to estimate the leaching of sulfentrazone from different treatments.

### 2.1. Reagents for the Laboratory Tests

The solvents used were High Performance Liquid Chromatography (HPLC) grade or analytical grade. Calcium chloride was supplied by Vetec, Brazil (>97%). Sulfentrazone [2′,4′-dichloro-5-(4-difluoromethyl-4,5-dihydro-3-methyl-5-oxo-1H-1,2,4-triazol-1-yl)phenyl)methanesulfonamide] (analytical standard, ≥92%), acetonitrile (≥99.9%) and ethyl acetate (≥99.7%) were obtained from Sigma-Aldrich, Darmstadt, Germany. A standard stock solution of 1000 mg L^−1^ sulfentrazone in acetonitrile was used to prepare the working solutions in 0.01 mol L^−1^ CaCl_2_ solution. The glyphosate formulations used were Roundup Ready^®^ (isopropylamine salt), Roundup Ultra^®^ (ammonium salt), and Zapp Qi^®^ (potassium salt), all of which were obtained from a local commercial pesticide center.

### 2.2. Soil Collecting

The soil used for the laboratory experiments (sorption, desorption, and extraction capacity of sulfentrazone) and in the greenhouse (leaching) was collected from the 0–20 cm depth layer. After collection, the soil was dried in the shade, sieved with a 2 mm mesh, and characterized chemically and physically (Table 1). The soil presented a sandy texture similar to that of the soils of midwest Brazil, a region that is commonly cultivated with soybean, which is subjected to glyphosate and sulfentrazone applications.

### 2.3. Sorption and Desorption Experiment of Sulfentrazone Alone and Mixed with Glyphosate Formulations

#### 2.3.1. Experimental Design

The experiment was carried out in a completely randomized design with three repetitions, and was replicated twice. The treatments were composed of the presence of sulfentrazone in isolation and mixed with three formulations of glyphosate (Roundup Ready^®^, Roundup Ultra^®^, and Zapp Qi^®^) in a sandy-textured soil.

#### 2.3.2. Determination of the Equilibrium Time

The determination of the equilibration time of the sulfentrazone in the soil was performed by the batch equilibrium method [15]. A solution was prepared to contain 20 mg L^−1^ sulfentrazone, obtained from a stock solution of 1000 mg L^−1^ sulfentrazone in 0.01 mol L^−1^ CaCl_2_. The solutions for the Roundup Ready^®^, Roundup Ultra^®^, and Zapp Qi^®^ formulations were prepared in a concentration of 10 mg L^−1^ N-(phosphonomethyl)glycine in 0.01 mol L^−1^ CaCl_2_. In 50 mL capacity tubes, 2.00 g of soil, 5 mL of sulfentrazone solution (20 mg L^−1^), and 5 mL of each formulation were added for each treatment. In the treatment with sulfentrazone alone, 5 mL of sulfentrazone solution (20 mg L^−1^) and 5 mL of 0.01 mol L^−1^ CaCl_2_ were added.

After this procedure, the tubes were sealed and agitated vertically for different amounts of time (0, 0.5, 1.0, 2.0, 4.0, 8.0, 12.0, 16.0, 24.0 and 28.0 h) at a controlled temperature (25 ± 2 °C) and 40 rpm. After stirring, the samples were centrifuged at 2260× *g* (3500 rpm) for seven minutes. Part of the supernatant was filtered through a 0.45 μm PTFE membrane (Millipore^®^ Darmstadt, Germany) and placed directly into a 1.5 mL vial for later analysis by high performance liquid chromatography (HPLC). The equilibrium time considered was that from which the concentration of the analyzed solution remained constant.

#### 2.3.3. Sulfentrazone Sorption

The sorption analysis was performed using working solutions from a stock solution of 1000 mg L^−1^ sulfentrazone (analytical standard, 92.01% purity) with concentrations of 1.0, 2.0, 5.0, 10.0, and 20.0 mg L^−1^ in 0.01 mol L^−1^ CaCl_2_. The solutions for the Roundup Ready^®^, Roundup Ultra^®^, and Zapp Qi^®^ formulations were prepared in a 10 mg L^−1^ N-(phosphonomethyl)glycine in 0.01 mol L^−1^ CaCl_2_. Into 50 mL capacity tubes were added 2.00 g soil, 5 mL sulfentrazone solution (varying with each concentration) and 5 mL of each formulation, according to each treatment proposed. In the treatment with sulfentrazone alone, 5 mL sulfentrazone solution (varying according to each concentration) and 5 mL 0.01 mol L^−1^ CaCl_2_ were added.

After this procedure, the tubes were sealed and agitated vertically at a controlled temperature (25 ± 2 °C) and 40 rpm for the equilibrium time previously determined. After stirring, the samples were centrifuged at 2260× *g* (3500 rpm) for seven minutes. A portion of the supernatant was filtered through a 0.45 µm membrane (Millipore^®^) and transferred to 1.5 mL vials for the HPLC analysis.

#### 2.3.4. Sulfentrazone Desorption

The desorption was performed immediately after the sorption at all points of the sorption isotherms. The supernatant from the sorption was completely removed, and the tubes remained at a controlled temperature (25 ± 2 °C) until the complete evaporation of the solvent. Then, 10 mL 0.01 mol L^−1^ CaCl_2_ whitout herbicide was added to all of the tubes. After this procedure, the tubes were agitated vertically at a controlled temperature (25 ± 2 °C) and 40 rpm. The suspensions were centrifuged at 2260× *g* (3500 rpm) for seven minutes, and the supernatants were also filtered through a 0.45 µm membrane (Millipore^®^) and analyzed by HPLC. The amount of herbicide still sorbed in the soils at each desorption step was calculated as the difference between the amount of the herbicide sorbed and the amount desorbed.

The concentration of the herbicide sorbed to the soil (Cs) in mg kg^−1^ was calculated from the difference between the standard solutions added to the soil (Cp) in mg L^−1^ and the amount found in the equilibrium solution (Ce) in mg L^−1^. The Freundlich equation was used (Cs = Kf * Ce^n^) to interpret the results of the sorption and desorption process based on the values of Cs and Ce. After the construction of the sorption and desorption isotherms, the hysteresis index was calculated by dividing the linearity coefficients ‘n’ of the sorption by ‘n’ of the desorption [16].

### 2.4. Leaching Experiment of Sulfentrazone Isolated and Mixed with Glyphosate Formulations

#### 2.4.1. Experimental Design

The experiment was carried out in a completely randomized design with four replicates. The treatments were arranged in subdivided plots, with the plots corresponding to the herbicides (Sulfentrazone, Sulfentrazone + Roundup Ready^®^, Sulfentrazone + Roundup Ultra^®^, and Sulfentrazone + Zapp Qi^®^) and the subplots corresponding to the sampling depths in the soil columns (0–5, 5–10, 10–15, 15–20, 20–25, 25–30, 35–40, 40–45 and 45–50 cm).

#### 2.4.2. Preparation of the Columns

The sulfentrazone leaching was estimated using PVC (polyvinyl chloride) columns that were 10 cm in diameter and 50 cm in length. First, the insides of the columns were coated with a layer of paraffin to prevent lateral water runoff. After this procedure, the columns were filled with 4.5 kg of the red-yellow oxisol. After the columns were filled, they were saturated with water for 48 h in order to eliminate possible air bubbles trapped in the pores. Subsequently, the columns were left upright for 72 h to drain any excess water, allowing the soil moisture to approximate the field capacity.

#### 2.4.3. Herbicide Application and Rain Simulation

The herbicides were applied to the top of the columns using a high-precision, CO_2_-pressurized sprayer (TTI—Turbo TeeJet Induction 110 02 nozzles, calibrated to apply 150 L ha^−1^). Sulfentrazone (Boral^®^—FMC, Campinas—SP—Brazil) was applied at a dose of 1500 g ha^−1^. In the treatments with the presence of the formulations, the glyphosate was applied at a dose of 1080 g ha^−1^ simultaneously with the sulfentrazone. The applications were carried out at 7:00 a.m. at a temperature of 25 ± 2 °C and a relative humidity of 70%, which are ideal conditions for pesticide applications. Twelve hours after the application of the herbicide, 60 mm of rain was simulated for two hours, with the columns upright. During the rain simulation, rain gauges were installed next to the columns in order to monitor the volume of water applied. The columns were then maintained in a vertical position for 72 h in order to drain any excess water. After this procedure, the columns were cut into 5 cm segments, totaling ten segments per column. The soil samples contained in each segment were homogenized and stored at −20 °C until the time of the chromatographic analyses.

#### 2.4.4. Method of Extraction for Sulfentrazone

The extraction of sulfentrazone from the soil for quantification was carried out using the low-temperature partitioning solid–liquid extraction technique (SLE/LTP, and analyzed by high-performance liquid chromatography (HPLC-UV/Vis) as described by Costa et al. (2015) [17], with modifications. In this methodology, 2 g dry soil, obtained from the samples of each segment of the columns, was weighed in Falcon tubes with a capacity of 50 mL, and 12 mL extraction solution was added (6.5 mL acetonitrile, 1.5 mL ethyl acetate and 4 mL water at pH 8). The tubes were vortexed for 2 min and then maintained at −20 °C for 4 h. The supernatant was filtered on qualitative filter paper containing 1 g anhydrous Na_2_SO_4_, and transferred to a 50 mL flask. The flask was coupled to a rotary evaporator and maintained at 40 °C and 100 rpm until the complete evaporation of the solvent. The residue contained in the flask was recovered in 1.5 mL acetonitrile, filtered through a Millipore filter (0.45 μm), and analyzed by HPLC-UV/Vis.

#### 2.4.5. Validation of the Quantification and Extraction Method

The optimized method for the determination of sulfentrazone in red-yellow oxisol in isolated applications and mixed with Roundup Ready^®^, Roundup Ultra^®^, and ZappQi^®^ was validated according to the criteria of selectivity, linearity, detection and quantification limits, accuracy and precision (repeatability and intermediate accuracy). The validation was carried out following the guidelines proposed by the national and international methods of the validation of chemical tests [18]. The results of the validation of the quantification and extraction method are presented in the Electronic Supplementary Material-1 (Figures S1–S5, Tables S1–S4) [19,20]

#### 2.4.6. Chromatographic Conditions

The sulfentrazone quantification was performed according to a method proposed by Ohmes et al. (1999) [21] and adapted by Passos et al. (2013) [22], utilizing a photodiode array detector (Shimadzu SPD-M20A) and a C18 stainless steel column (Shimadzu VP-ODS Shim-pack 250 mm × 4, 6 mm id, 5 μm particle size).

The chromatographic conditions were as follows: a mobile phase consisting of acetonitrile:water (acidified with 0.1% orthophosphoric acid) at a ratio of 50:50, an injection volume of 20 μL, a flow of 1 mL min^−1^, a wavelength of 207 nm, and a column temperature of 30 °C. Under these conditions, the retention time for the sulfentrazone was approximately 7.2 min. A comparison of the sample retention time with an analytical sulfentrazone standard permitted the identification of the herbicide, and its quantification was determined by the external calibration method.

### 2.5. Statistical Analysis of the Experiments

The generated data were submitted to the Shapiro–Wilk residual normality test [23]. The Bartlett test was used to test the homoscedasticity of the data [24]. The sorption and desorption coefficients (Kfs and Kfd), and the hysteresis index were used to compare the sorption and desorption capacity of the sulfentrazone in the red-yellow oxisol in the different treatments. The confidence interval of the mean, to a *p*-value of 0.05, was used for the comparison of the sulfentrazone concentration in each depth segment. All of the graphics were created using the SigmaPlot 12.0 program (Exact Graphs and Data Analysis) for Windows.

## 3. Results and Discussion

### 3.1. Adsorption Kinetics of Sulfentrazone Isolated and Mixed with Glyphosate Formulations

In order to understand the sorption kinetics of sulfentrazone in the soil, we used and compared two models: pseudo-first-order and pseudo-second-order. The pseudo-first-order is described by the following equation: ln (qe − qt) = ln(qe) − *k_1_*t, where qe is the amount of the compound absorbed at the equilibrium time, qt is the amount of the compound absorbed at time t, and *k_1_* is the constant of the sorption rate (min^−1^). The second model is described by the following equation: (t/qt) = (t/qe) + [1/(*k_2_* qe^2^)], where qe is the amount of the compound absorbed at the equilibrium time, qt is the amount of the compound absorbed at time t, and *k_2_* is the constant of the sorption rate (g mg^−1^ min^−1^) [25].

The nonlinear pseudo-first-order and pseudo-second-order models presented a high R^2^ for all of the treatments, varying between 0.96 to 0.99 (Table 2). The parameters qe and k for the pseudo-first-order and pseudo-second-order models were significant at *p*-value ≤ 0.05 (Table 2). The pseudo-first-order was slightly better-adjusted than the pseudo-second order. For the evaluation of the differences among the applications of sulfentrazone alone and mixed with glyphosate formulations, the pseudo-first-order was chosen.

**Figure 1:**
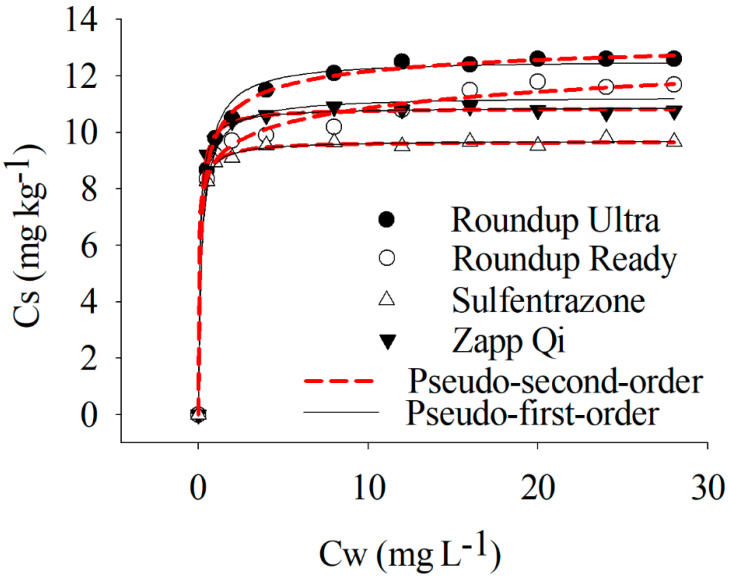
Estimates of the adsorption kinetics curve for sulfentrazone alone and mixed with glyphosate formulations.

The good adjustment of the kinetic models allowed us to understand the adsorption of the pesticides to the soil correctly. For sulfentrazone, both isolated and mixed, the first-order nonlinear model showed better performance in explaining the adsorption kinetics of this herbicide. Sorbates that exhibit high affinity to an adsorbate fit better with pseudo-first-order models due to the exponential adsorption early in the exposure periods. This behavior was observed for sulfentrazone alone and mixed in the studied soil (Figure 1). In this work, 80% (the mean value of the treatments) of the herbicide molecules were adsorbed by the soil at 54.1, 28.3, 29.2, and 30.5 min for sulfentrazone, sulfentrazone + Roundup Ready^®^, sulfentrazone + Roundup Ultra^®^, and sulfentrazone + Zapp Qi^®^, respectively.

The presence of the glyphosate formulations did not alter the equilibrium time of sulfentrazone (Figure 1). The time required for the balance between the amount of sulfentrazone sorbed to the soil and present in the solution was 4 h for all of the treatments (Figure 1). Initially, sulfentrazone was rapidly adsorbed by organic and mineral colloids in the soil, with the adsorption of 8.5 mg kg^−1^ of the herbicide 0.5 h after the beginning of the agitation of the tubes containing the soil and 10 mg L^−1^ sulfentrazone. This higher initial adsorption to the soil was observed for different organic pesticides due to the high availability of sites capable of adsorbing organic molecules [26,27,28]. Passos et al. (2013) [22] also reported a similar behavior for sulfentrazone in different Brazilian soils after the first few minutes of exposure between the herbicide and the soil.

After the exponential adsorption of sulfentrazone, a second phase is initiated with slower sorption of the herbicide to the soil. The occupation of the sites available for the adsorption of sulfentrazone and the repulsion promoted by the absorbed herbicide hamper the sorption of new molecules of the herbicide present in the solution, reducing the adsorption rate [29,30]. Despite the slow process, sulfentrazone continued to be adsorbed to the soil for four hours under stirring. At that point, a third phase was initiated, and the herbicide could not bind to the soil. The third phase is when the concentrations between the amount sorbed and in the solution become constant [22]. For the sulfentrazone alone and mixed with the glyphosate formulations, this equilibrium was reached after four hours. A period of six hours was chosen in order to ensure equilibrium between the sulfentrazone concentrations in the soil and the solution.

### 3.2. Sorption and Desorption Isotherms of Sulfentrazone Alone and Mixed with Glyphosate Formulations

**Figure 2:**
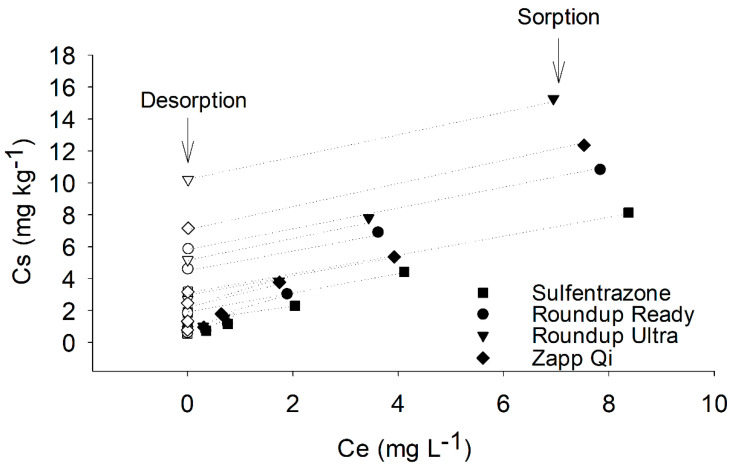
Sorption and desorption isotherms adjusted to the Freundlich model for sulfentrazone alone and mixed with glyphosate formulation.

The Freundlich isotherm was adjusted to describe the sulfentrazone’s sorption alone and mixed with glyphosate formulations (Figure 2). The RSME (Root Mean Square Error) values ranged from 0.03 to 0.08, and the Kfs values ranged from 1.34 to 2.34 (Table 3).

The values of Kfs obtained were similar to those observed in another study that evaluated the adsorption capacity of sulfentrazone in Brazilian soils [22]. The isotherms had a high adjustment, and allowed the comparison of the sorption capacity of isolated and mixed sulfentrazone through the sorption coefficient obtained for each treatment.

The presence of the formulated Zapp Qi^®^, Roundup Ready^®^, and Roundup Ultra^®^ products caused increases in the Kfs values compared to that with sulfentrazone alone (Table 3). Higher Kfs values indicate that the formulations increased the sulfentrazone sorption to the soil. Therefore, it is evident that the glyphosate or other inert ingredients that make up the formulated product affected the interaction between the sulfentrazone molecule and the soil, altering the natural retention process of sulfentrazone to the soil. Rd allows the classification of the adsorption of compounds in binary systems in three cases: (i) if the Rd > 1, there is synergism; (ii) if the Rd < 1, there is antagonism; and (iii) if the Rd ≈ 1, there is no interaction [31]. For the adsorption of sulfentrazone to the soil in binary systems, it is possible to observe that the glyphosate formulations acted as synergists, with Rd values equivalent to 1.46, 1.65, and 1.76 for the Zapp Qi^®^, Roundup Ready^®^, and Roundup Ultra^®^ formulations, respectively (Table 3).

There are no reports on the role of glyphosate formulations in increasing sulfentrazone sorption to the soil. Despite the synergistic effect between the sulfentrazone and glyphosate formulations, this behavior was not observed for other herbicide mixtures. For example, Mendes et al. (2018) [32] did not note differences in mesotrione sorption in seven Brazilian soils when it was applied alone or mixed with S-metolachlor.

In the soil solution, the phosphate group of the glyphosate molecule can be rapidly sorbed to the iron and aluminum oxides present in clay colloids [33]. Once it is adsorbed to the soil, glyphosate can increase the sorption of sulfentrazone due to the presence of chemical groups in both molecules that allow the establishment of hydrogen bonds between them. These groups can be identified when their molecular structures are evaluated (Electronic Supplementary Material-2 ). Although sulfentrazone also binds to iron and aluminum oxides due to its zwitterionic ion behavior in neutral form [34,35,36], the sulfentrazone is adsorbed more slowly than glyphosate, favoring the formation of a soil-glyphosate-sulfentrazone complex. This interaction between glyphosate and sulfentrazone would be possible since sulfentrazone is adsorbed more slowly than glyphosate, forming a soil-glyphosate-sulfentrazone complex. The possible hydrogen bonds between glyphosate and sulfentrazone may also explain the difference observed for the Kfs values of sulfentrazone mixed with glyphosate formulations.

The Roundup Ultra formulation presents the lowest molecular weight, and 10 hydrogen donors and acceptors (Table 4). The lower molecular weight of glyphosate in the Ultra formulation may allow a higher number of molecules to bind to the soil than other formulations. The lower molecular weight of a pesticide has already been associated with higher soil sorption [37]. Therefore, the greater number of the glyphosate molecules of the Ultra formulation bound to the soil can increase sulfentrazone sorption via hydrogen bonds, explaining the higher Kfs value.

Different behaviors were observed when comparing the Ready and Zapp Qi formulations. The Ready formulation had the highest molecular weight, and the sulfentrazone sorption for this formulation was higher than that for Zapp Qi (Table 3 and Table 4). This fact may be related to the lower number of sites in the Zapp Qi formulation to establish hydrogen bonds (9) compared to the number in Ready (10), reducing the sulfentrazone adsorption. Radian et al. (2015) [37] correlated the physicochemical properties of several pesticides with their soil sorption, and showed that the molecule with the highest number of hydrogen donor and acceptor sites has a high correlation with the sorption of these organic compounds.

The increase in the sorption of sulfentrazone when mixed with the Ultra^®^, Ready^®^, and Zapp Qi^®^ formulations may reduce the mobility of this herbicide in soils, and consequently the risk of leaching and surface runoff [38,39]. From an environmental point of view, mixed applications have been shown to be safer than isolated sulfentrazone applications. However, it is essential to evaluate this herbicide’s desorption process under the conditions of isolated and mixed use for a better understanding of its mobility in the soil [40].

The Freundlich models obtained for the desorption of sulfentrazone alone and mixed with glyphosate formulations demonstrate a low RSME value (0.03 to 0.07), and the parameters of Kfd and ‘n’ were significant for the proposed model (Table 5). The good fit of the Freundlich model for desorption, similarly to sorption, indicates that the coefficients Kfd and ‘n’ can be used to explain the behavior of sulfentrazone in the soil.

The presence of glyphosate formulations increased the Kfd values compared to that with sulfentrazone alone (Table 5). The Kfd values for sulfentrazone were 1.9-, 3.7-, and 11.2-fold greater in the Ready, Zapp Qi, and Ultra formulations, respectively, than in the isolated treatment (Table 5). The Kfd value refers to the herbicide’s ability to remain sorbed to the soil after shaking in the extractive solution. Therefore, soils with high Kfd values have a lower desorption capacity for an herbicide [41]. The presence of the formulated products reduced the sulfentrazone desorption, and this phenomenon may be associated with a higher affinity of sulfentrazone to the soil in the presence of glyphosate, as observed in the sorption tests. The electrostatic and hydrogen bonds that may have occurred between the soil-glyphosate-sulfentrazone complexes may have increased the bonds’ stability, requiring higher energy for removal of the sulfentrazone absorbed.

The Ultra formulation provided the lowest desorption and the highest sulfentrazone sorption to the soil. This fact indicates that the interactions occurring between sulfentrazone and the soil in the presence of this formulation are stable, and that the presence of the Ultra formulation does not increase the horizontal and vertical mobility of this herbicide. However, it was possible to observe that the sulfentrazone and Ready formulation showed greater desorption than that of the treatment mixed with Zapp Qi, even allowing the higher initial sorption of sulfentrazone.

The Ultra and Zapp Qi formulations have a higher number of rotational bonds than that in the Ready formulation; as such, after establishing the interactions between glyphosate and sulfentrazone, the glyphosate molecules from the Ultra and Zapp Qi formulations can more easily exhibit intramolecular rotations. Some work has demonstrated the role of rotational bonds in increasing the complexity and crystallization of molecules, indicating that this feature may increase the stability of interactions between organic molecules [42,43,44,45]. Thus, this mechanism may have reduced the amount of sulfentrazone desorbed in the Ultra and Zapp Qi formulations relative to the Ready formulation.

The hysteresis index for the treatments ranged from 0.88 to 0.90 (Table 5). According to Bariusso et al. (1994) [46], the values for the hysteresis index—ranging from 0.7 to 1.0—indicate that it is not possible to confirm the existence of this phenomenon. This fact was observed for sulfentrazone independent of the applied treatment, so it is possible to conclude that the sorption and desorption processes occur with the same intensity in this work’s conditions.

The evaluation of the sorption and desorption of sulfentrazone alone and mixed with glyphosate showed that these processes were altered and could affect the dynamics of this pesticide in soils. The lower sorption and higher desorption of the herbicide in the soil increase the risk of the contamination of freshwater and groundwater, and thus use under these conditions should be avoided [39]. The presence of the glyphosate formulations increased the sorption and reduced the desorption of sulfentrazone compared to its application alone, so according to these parameters, the risk of environmental contamination did not increase due to the mixing of these products. However, assays that estimate the leaching of herbicides in the soil should be carried out in order to confirm these results. Among the methods used to evaluate leaching, simulation in columns is a good way to quantify the leaching potential of pesticides [47,48].

### 3.3. Estimated Leaching of Sulfentrazone Applied Alone and Mixed with Glyphosate Formulations

The sulfentrazone leaching in the different treatments was evaluated in 50 cm depth columns. However, the sulfentrazone was only detected and quantified up to 20–25 cm deep (Table 6). The isolated sulfentrazone reached the 10–15 cm layer on the columns with a concentration of 0.35 mg kg^−1^ (Table 6). The Ultra formulation reduced the sulfentrazone leaching compared to that of the other treatments, with quantification only until a depth of 10 cm (Table 6). The highest concentration of sulfentrazone was observed when combined with the Ultra formulation at a concentration of 1.25 mg kg^−1^ (Table 6).

Theoretically, increasing the sorption and reducing the desorption of an herbicide reduces the molecule’s mobility, preventing it from leaching to deeper layers of soil [48,49]. This effect was observed for sulfentrazone when mixed with Ultra. In this treatment, higher sorption and lower desorption were observed, reflecting lower sulfentrazone leaching. Environmentally, this behavior is favorable, since it is possible to reduce the risk of sulfentrazone leaching, ensuring a safer application of this pesticide [31].

The highest leaching of sulfentrazone was observed in the treatment with the Ready formulation, reaching the 20–25 cm depth layer (Table 6). Unlike Ultra, the increase in sorption and reduction in sulfentrazone desorption promoted by the presence of the Ready formulation did not cause any less leaching of the molecule. This behavior observed for Ready seems contradictory; however, more leaching may be observed in conditions of the greater sorption and lower desorption of a pesticide. The Ready formulation delayed the sulfentrazone adsorption process in the soil compared to the process in the other treatments. The K_1_ and K_2_ values for sulfentrazone + Roundup Ready^®^ (2.75 and 1.67, respectively) were lower than those for sulfentrazone alone and mixed with other formulations (Table 2). The constants K_1_ and K_2_ indirectly indicate the sorption rate of sorbets to adsorbents [25]. Consequently, more sulfentrazone may still be in the soil solution at the time of the rain simulation when it is mixed with the Ready formulation. This fact raises the probability of herbicide leaching, as observed for sulfentrazone mixed with Roundup Ready.

Khan and Brown (2017) [49] reported a similar effect for propyzamide. The authors observed higher leaching of the formulated product propyzamide compared to that for the same herbicide in its pure form, even with the formulated product exhibiting increased sorption. The authors attributed the increased leaching of the formulated product to the surfactants that can remain associated with the active substance, retarding the sorption process and thus increasing the leaching.

The sulfentrazone initially sorbed to the soil when it was combined with Ready formulation may initially reduce the number of available molecules in the soil solution. However, when the adsorption is not of high stability, the herbicide can gradually return to the soil solution and be leached after rainfall. Reddy and Locke (1998) [50] demonstrated that the higher sorption of sulfentrazone in no-tillage soils compared to conventional tillage soils may prolong sulfentrazone persistence due to the lower amount of herbicide available in the soil. However, sulfentrazone was able to return to the soil solution, increasing the amount available for leaching. Evidence that may support this behavior is the higher total amount detected (in the sum of all layers) of sulfentrazone (1.5-fold higher) in the sulfentrazone + Roundup Ready^®^ treatment compared to the sulfentrazone treatment alone (Table 6). The lower sorption observed when sulfentrazone was applied alone may allow the degradation of the herbicide in the first few hours, reducing the amount of sulfentrazone leached into the soil profile.

Although the simulations using PVC columns do not reflect the real scenario in agricultural fields, the results demonstrate a perspective of the risks involved in the mixed application of these herbicides. When applied in a mixture with Zapp Qi^®^, these results showed that the leaching of sulfentrazone reached a depth similar to that of sulfentrazone alone (Table 6). However, a larger total amount was quantified in the sulfentrazone + Zapp Qi^®^ treatment than with sulfentrazone alone (Table 6). Similar to that in the Ready formulation, the higher sulfentrazone sorption in the presence of Zapp Qi^®^ reduced the amount of the herbicide in the soil solution that was available for degradation, but the lowest desorption rate in the sulfentrazone + Zapp Qi^®^ treatment (2-fold higher than that in the sulfentrazone + Ready treatment) minimized the effects of rainfall on the sulfentrazone leaching.

## 4. Conclusions

The experiments demonstrated that the sorption and desorption processes for sulfentrazone combined with other formulated glyphosate products are altered, supporting the hypothesis raised in this work that the presence of other molecules is a factor that affects the behavior of herbicides in the soil. The change in the sulfentrazone sorption and desorption to the soil when it was mixed affected its leaching. The use of sulfentrazone mixed with Roundup Ultra formulation has been shown to be safer, reducing the leaching of the product and consequently the potential for the contamination of groundwater. In contrast, the Roundup Ready formulation promotes increased sulfentrazone leaching, and therefore this practice should be avoided in regions with high rainfall and sandy soils. Therefore, situations where pesticides are combined during the application should be evaluated in order to improve the understanding of these molecules’ dynamics and avoid environmental contamination.

## Figures and Tables

**Table 1:** Physical and chemical parameters of the red-yellow oxisol used in the experiment.

Sand (%)				Silt (%)				Clay (%)		
76				7				17		
pH	P	K^+^	Ca²^+^		Mg²^+^	Al^3+^	H + Al	SB	CEC (t)	OM
H_2_O	--------------------------------------- cmol_c_ dm^−3^ ---------------------------------------------------------									%
4.7	2.3	41	2.2		0.7	0.2	5.61	3.0	3.2	2.5

Analyses carried out in the Soil Analysis Laboratory of Viçosa, according to the methodology of the Brazilian Agricultural Research Company—EMBRAPA (1997) [14]; SB: sum of bases; CEC: cation exchange capacity; OM: organic matter.

**Table 2:** Parameters of the pseudo-first order and pseudo-second order models for the adsorption of sulfentrazone in isolation and mixed with glyphosate formulations.

Kinetic Model	Parameters			
	Sulfentrazone	Sulfentrazone + Roundup Ready^®^	Sulfentrazone + Roundup Ultra^®^	Sulfentrazone + Zapp Qi^®^
Pseudo 1st order	** qe: 9.63 mg	** qe: 11.67 mg**	*** qe: 12.98 mg	** qe: 10.86 mg
	* k_1_: 4.24 min^−1^	*** *k*_1_: 2.70 min^−1^	** *k*_1_: 4.02 min^−1^	** *k*_1_: 4.17 min^−1^
	*R*^2^: 0.99	*R*^2^: 0.99	*R*^2^: 0.97	*R*^2^: 0.99
Pseudo 2nd order	* qe: 9.22 mg	** qe: 11.02 mg	** qe: 12.92 mg	** qe: 10.33
	** *k*_2_: 2.06 g mg^−1^ min	** *k*_2_: 1.67 g mg^−1^ min	** *k*_2_: 1.92 g mg^−1^ min	*** *k*_2_: 2.04 g mg^−1^ min
	*R*^2^: 0.96	*R*^2^: 0.96	*R*^2^: 0.99	*R*^2^: 0.99

Significant values: * *p* < 0.05, ** *p* < 0.01, *** *p* < 0.001.

**Table 3:** Parameters of the sorption isotherms and ratio adsorption capacities (Rd) for sulfentrazone in isolation and mixed with glyphosate formulations.

Herbicides	Parameters	Estimative	Std. Error	^a^ RMSE	Rd
Sulfentrazone	Kfs	1.3 ***	±0.1	0.05	-
	1/n	0.8 ***	±0.1		
Sulfentrazone + Roundup Ready^®^	Kfs	2.1 *	±0.4	0.06	1.65
	1/n	0.8 **	±0.1		
Sulfentrazone + Roundup Ultra^®^	Kfs	2.3 ***	±0.1	0.05	1.76
	1/n	1.0 ***	±0.0		
Sulfentrazone + Zapp Qi^®^	Kfs	1.9 *	±0.5	0.03	1.46
	1/n	0.9 **	±0.1		

^a^ RSME = Root mean square error. Significant values: * *p* < 0.05, ** *p* < 0.01, *** *p* < 0.001, - Not applicable.

**Table 4:** Physical and chemical properties of glyphosate formulations.

Property Name	Formulations		
	Roundup Ultra	Roundup Ready	Zapp Qi
Salt	Glyphosate ammonium	Isopropylamine salt	Potassium
Molecular weight (g mol^−1^)	186.1	346.4	207.1
Hydrogen bond donor count	4	4	3
Hydrogen bond acceptor count	6	6	6
Rotatable bond count	4	2	4

**Table 5:** Parameters of the desorption isotherms and hysteresis index (H) for sulfentrazone in isolation and mixed with glyphosate formulations.

	Parameters	Estimative	Std. Error	^a^ RSME	H
Sulfentrazone	Kfd	65.7 **	5.3	0.03	0.88
	1/n	0.7 *	0.1		
Sulfentrazone + Roundup Ready	Kfd	125.2 *	8.7	0.06	0.88
	1/n	0.7 *	0.1		
Sulfentrazone + Roundup Ultra	Kfd	733.3 ***	6.4	0.07	0.90
	1/n	0.9 **	0.1		
Sulfentrazone + Zapp Qi	Kfd	239.8 *	4.2	0.04	0.89
	1/n	0.8 *	0.1		

^a^ RSME = root mean square error. Significant values: * *p* < 0.05, ** *p* < 0.01, *** *p* < 0.001.

**Table 6:** Sulfentrazone concentrations (mean ± interval of confidence) in the column profile submitted to sulfentrazone application in isolation and mixed with formulations of glyphosate: Roundup Ready^®^, Roundup Ultra^®^, and ZappQi^®^.

Depth	Sulfentrazone	Sulfentrazone + Roundup Ready	Sulfentrazone + Roundup Ultra	Sulfentrazone + Zapp Qi
	mg kg^−1^			
0–5	0.62 ± 0.04	1.10 ± 0.04	1.25 ± 0.04	0.92 ± 0.03
5–10	0.50 ± 0.03	0.55 ± 0.02	0.25 ± 0.04	0.60 ± 0.02
10–15	0.35 ± 0.02	0.15 ± 0.02	-	0.35 ± 0.02
15–20	-	0.20 ± 0.02	-	-
20–25	-	0.20 ± 0.02	-	-
Total	1.47 ± 0.03	2.20 ± 0.02	1.5 ± 0.04	1.87 ± 0.03

- Herbicide not detected.

## Supplementary Materials

The supplementary materials are available online at https://www.mdpi.com/2305-6304/8/4/123/s1. Figure S1: Chromatograms of soil extracts, obtained by the ESL / PBT-CLAE-UV / Vis method, fortified with 2 mg L-1 with sulfentrazone in an isolated application (A) and in a mixture with Roundup Ready^®^ (B), Roundup Ultra ^®^ (C), Zapp Qi^®^ (D) and herbicide-free soil (E); Figure S2. Sulfentrazone curve in acetonitrile; Figure S3. Matrix superimposed curves for sulfentrazone; Figure S4. Dispersion of analytical response regression as a function of isolated sulfentrazone concentration (A) and in mixture with Roundup Ready^®^ (B), Roundup Ultra^®^ (C) and ZappQi^®^ (D) herbicides, Table S1. Limits of detection and quantification of the proposed method in mg kg^−1^; Table S2. Percentages of recovery (% R) and coefficients of variation (% CV) obtained by the analysis of the extracts of the samples of the fortified Red-Yellow Oxisol, in three levels of concentration of sulfentrazone alone and in mixture with formulations of glyphosate. Table S3. Average peak area of the analytes in the chromatograms and coefficients of variation (CV) obtained in the analyzes of the samples of Red-Yellow Oxisol submitted to application of sulfentrazone alone and in mixture with Roundup Ready^®^, Roundup Ultra^®^ and Zapp Qi ^®^. Fortified samples were obtained at three levels of concentration, with six replicates at each level; Table S4. Concentration of sulfentrazone in the soil and coefficients of variation (CV) obtained in the analyzes of the fortified Red-Yellow Oxisol samples at three levels of concentration, with three replicates at each level, for the different days of analysis; Figure S5. Molecular structures of salts of ammonium (A), isopropylamine (B) and potassium (C), and sulfentrazone (D).
