# Supplementary material for: Influence of Glyphosate Formulations on the Behavior of Sulfentrazone in Soil in Mixed Applications
Source: Toxics. 2020 Dec 17;8(4):123. doi: 10.3390/toxics8040123 (PMC7766316; doi:10.3390/toxics8040123)
Supplement: Supplementary file 1 [file toxics-08-00123-s001.pdf]

Supplementary Materials: Influence of Glyphosate Formulations on the Behavior of Sulfentrazone in Soil in Mixed Applications

Ana Cláudia Langaro, Matheus de Freitas Souza, Gustavo Antônio Mendes Pereira, João Pedro Ambrósio Barros, Antonio Alberto da Silva, Daniel Valadão Silva, Ana Beatriz Rocha de Jesus Passos and Vander Mendonça

1. Electronic Supplementary Material-1

Validation of methodology to detection, quantification and extraction of sulfentrazone from the soil.

1.1. Selectivity

The selectivity of an instrumental method of separation is the ability to unequivocally evaluate the substances under examination in the presence of components that may interfere with their determination in a complex sample [19]. This fact was verified by the chromatograms of the soil matrix extracts, in the presence of the analytes of interest and the absence of them, obtained by the application of the ESL/PBT-CLAE-UV/V is method. The chromatograms obtained can be seen in Figure S1.

Selectivity evaluates the degree of interference of species as another active ingredient, excipients, impurities and degradation products, as well as other compounds of similar properties that may be present. Selectivity ensures that the peak response is exclusive of the compound of interest. If selectivity is not ensured, linearity, accuracy, and precision will be severely compromised [19].

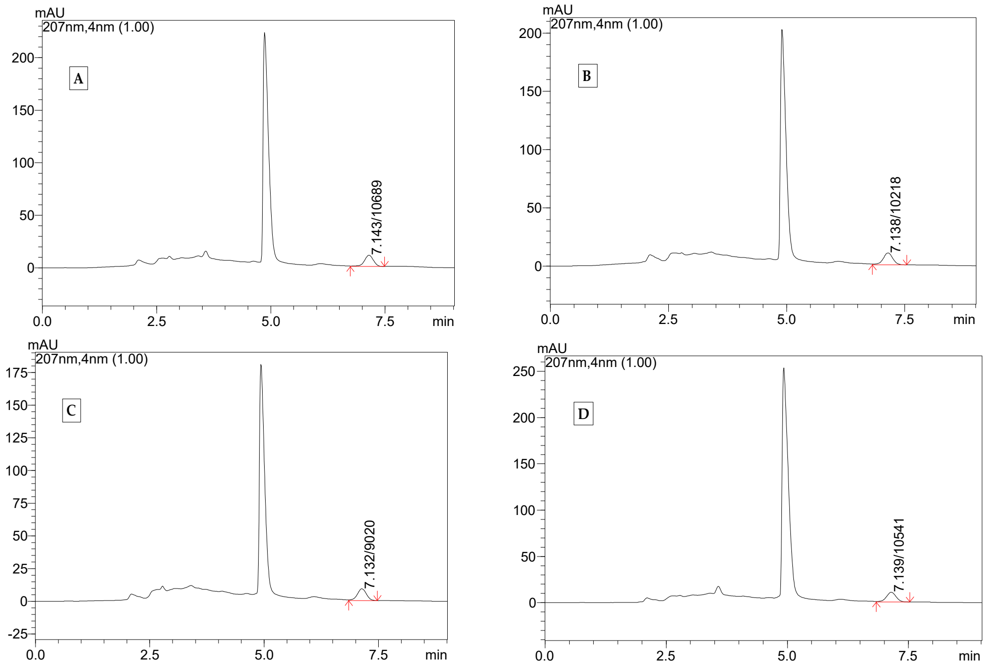

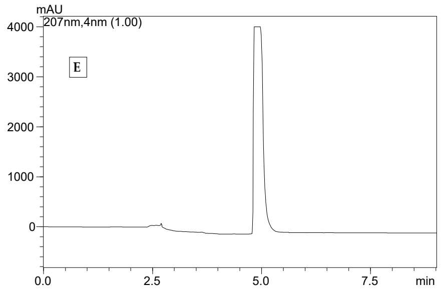

Figure S1. Chromatograms of soil extracts, obtained by the ESL / PBT-CLAE-UV / Vis method, fortified with 2 mg L-1 with sulfentrazone in an isolated application (**A**) and in a mixture with Roundup Ready® (**B**), Roundup Ultra ® (**C**), Zapp Qi® (**D**) and herbicide-free soil (**E**).

1.2. Linearity

Linearity corresponds to the ability of the method to provide results directly proportional to the concentration of the test substance within a given range of application. This was determined by injecting the extracts from the fortified samples into nine different concentration levels of the sulfentrazone herbicide applied in isolation and mixed with glyphosate formulations. The analytical curves obtained for the linearity of the equipment and the method of extraction of the soil herbicide are presented in Figures S2 and S3, respectively.

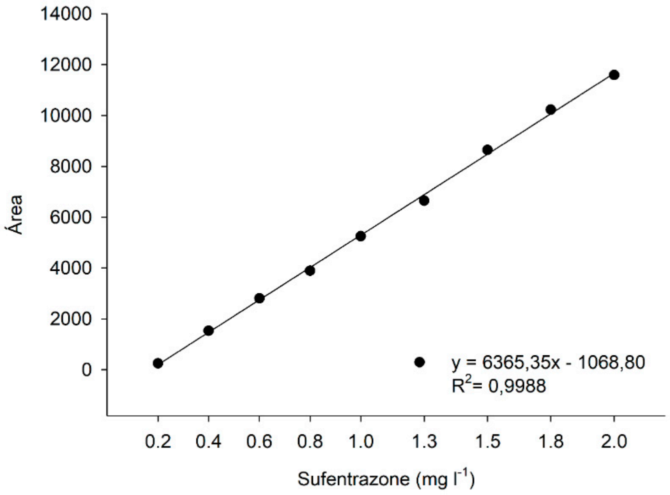

Figure S2. Sulfentrazone curve in acetonitrile.

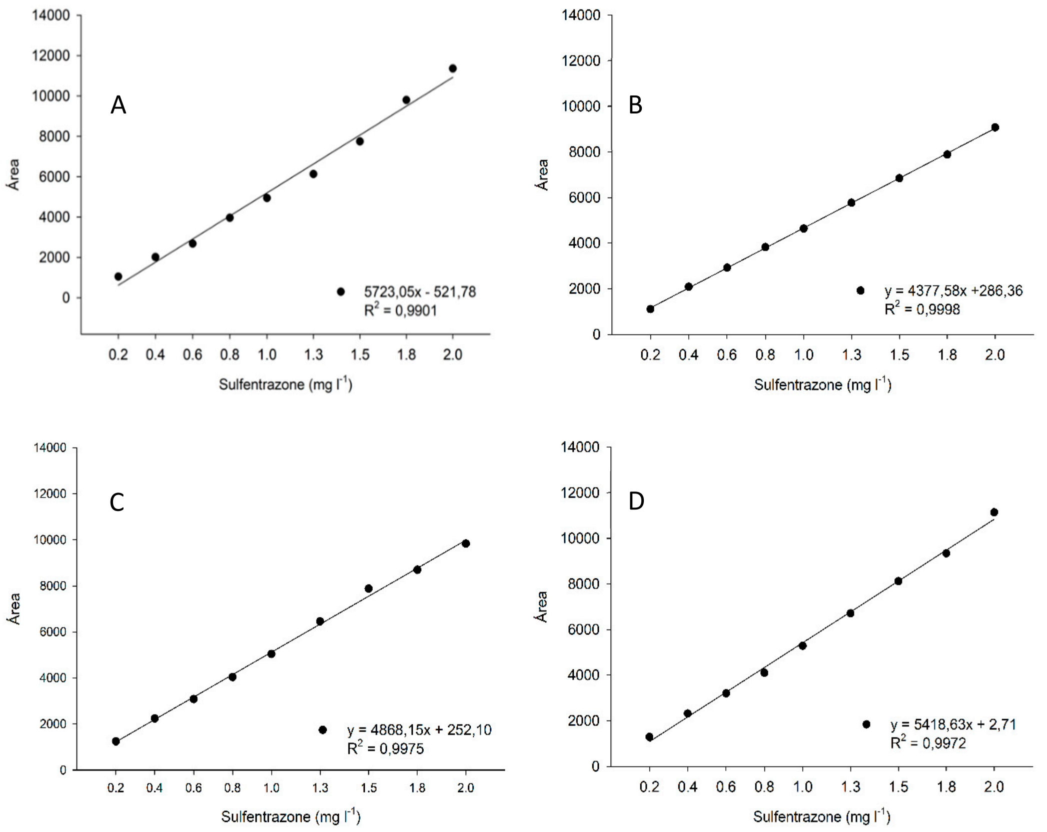

Figure S3. Matrix superimposed curves for sulfentrazone (**A**), sulfentrazone + Roundup Ready® (**B**), sulfentrazone + Roundup Ultra® (**C**) and sulfentrazone + ZappQi® (**D**) herbicides.

The estimation of the coefficients of an analytical curve from a set of experimental measurements can be done using the mathematical method known as linear regression. In addition to the regression coefficients a and b, it is also possible to calculate, from the experimental points, the coefficient of determination (R^2). This parameter allows an estimation of the quality of the obtained curve, because the closer to 1.0, the less the dispersion of the set of experimental points and the less the uncertainty of the estimated regression coefficients (Table S4).

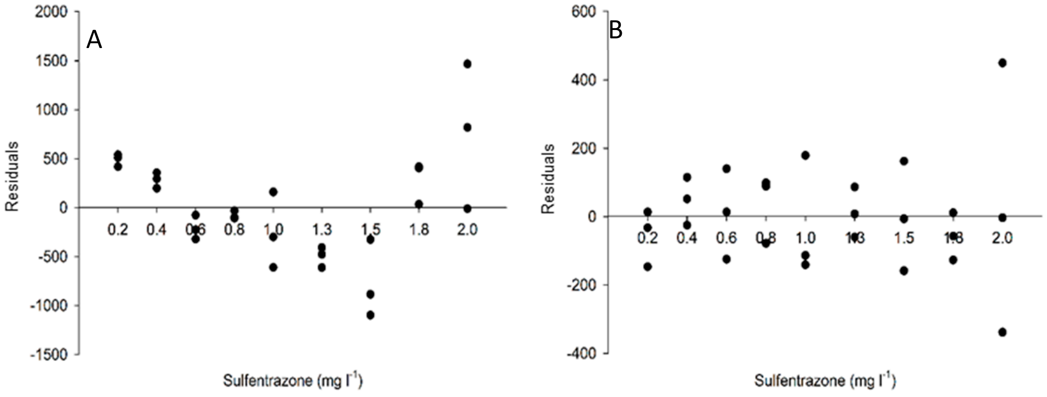

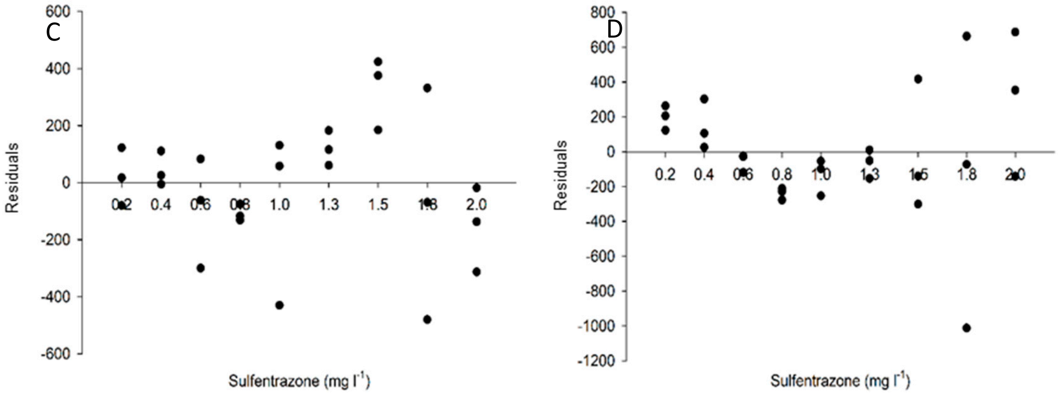

Figure S4. Dispersion of analytical response regression as a function of isolated sulfentrazone concentration (**A**) and in mixture with Roundup Ready® (**B**), Roundup Ultra® (**C**) and ZappQi® (**D**) herbicides.

The data set presented a random distribution for sulfentrazone in isolated and mixed applications with formulations of glyphosate. This distribution characterizes a homoscedastic behavior, besides indicating a good fit of the data to the model.

1.3. Detection and Quantification Limits

The detection (LOD) and quantification (LOQ) limits of the analytical curves of the herbicides in the different matrices were determined through equations 1 and 2 and are available in Table S1.

The detection limit (LOD) corresponds to the lowest concentration of the substance of interest that can be detected but not necessarily quantified accurately. The quantification limit (LOQ) corresponds to the lowest concentration of the substance of interest that can be accurately quantified [20].

Table S1. Limits of detection and quantification of the proposed method in mg kg^{-1}.

Herbicide	LOD (mg kg^{-1})	LOQ (mg kg^{-1})
Sulfentrazone	0.09	0.27
Sulfentrazone + Roundup Ready®	0.05	0.13
Sulfentrazone + Roundup Ultra®	0.06	0.18
Sulfentrazone + Zapp Qi®	0.08	0.25

1.4. Accuracy

To evaluate the accuracy, soil samples were fortified at three levels of sulfentrazone concentration in isolation and mixed with glyphosate and then submitted to the ESL/PBT-CLAE-UV/Vis method. The results obtained for recovery of the herbicide, together with its coefficients of variation, are shown in Table S2.

The % R values obtained ranged from 71.59% to 109.56%, with coefficient of variation from 0.75% to 13.57%. These results indicate that% R for both sulfentrazone in single application and in admixture with different glyphosate formulations are in accordance with the requirements of the validation guides, which report that "acceptable recovery ranges for residue analysis are generally between 70% and 120%, with accuracy up to ± 20%” [19].

Table S2. Percentages of recovery (% R) and coefficients of variation (% CV) obtained by the analysis of the extracts of the samples of the fortified Red-Yellow Oxisol, in three levels of concentration of sulfentrazone alone and in mixture with formulations of glyphosate.

Applied Concentration (mg L^{-1})	Concentration in the Soil (mg kg^{-1})	% R	CV (%)
	**Sulfentrazone**		
0.2	0.17	85.94	1.49
1.0	0.79	78.70	5.20
2.0	1.43	71.59	7.50
	**Sulfentrazone + Roundup Ready®**		
0.2	0.20	100.38	1.32
1.0	0.74	74.09	13.57
2.0	1.41	70.28	4.68
	**Sulfentrazone + Roundup Ultra®**		
0.2	0.20	102.87	4.08
1.0	0.74	73.81	1.87
2.0	1.45	72.28	4.45
	**Sulfentrazone + ZappQi®**		
0.2	0.22	109.56	7.40
1.0	0.76	75.65	5.24
2.0	1.48	73.78	0.75

1.5. Repeatability

Precision represents the dispersion of results between independent, repeated assays of the same sample, similar samples or standards, under defined conditions [19]. It can be estimated by relative standard deviation (DPR), also known as the coefficient of variation (CV). In methods of trace or impurity analysis, DPRs of up to 20% are accepted, depending on the complexity of the sample. The accuracy in method validation can be verified through repeatability and intermediate accuracy.

Repeatability represents the agreement between the results of successive measurements of the same method, carried out under the same measurement conditions. The values for repeatability are shown in Table S3.

Table S3. Average peak area of the analytes in the chromatograms and coefficients of variation (CV) obtained in the analyzes of the samples of Red-Yellow Oxisol submitted to application of sulfentrazone alone and in mixture with Roundup Ready®, Roundup Ultra® and Zapp Qi ®. Fortified samples were obtained at three levels of concentration, with six replicates at each level.

Applied Concentration (mg L^{-1})	Peak Area	CV (%)
	**Sulfentrazone**	
0.2	0.16	9.89
1.0	0.61	6.27
2.0	1.26	4.71
	**Sulfentrazone + Roundup Ready®**	
0.2	0.17	10.67
1.0	0.64	4.90
2.0	1.16	4.09
	**Sulfentrazone + Roundup Ultra®**	
0.2	0.17	10.09
1.0	0.63	6.08

2.0	1.13	7.90
	**Sulfentrazone + ZappQi®**	
0.2	0.17	6.87
1.0	0.62	7.00
2.0	1.19	4.04

The data obtained indicate a concordance between the results obtained by the application of the ESL/PBT-CLAE/UV-Vis method for both sulfentrazone in an isolated application and in a mixture with glyphosate. The coefficients of variation were between 4 and 10%, being below 20%, which is the accepted value for complex samples [43].

1.6. Intermediate Accuracy

Intermediate precision indicates the effect of variations within the laboratory due to events such as different days or different analysts or different equipment or a combination of these factors (Table S4). It is recognized as the most representative of the variability of the results in a single laboratory. The objective of the validation of the intermediate precision is to verify that in the same laboratory the method will provide the same results [19].

Table S4. Concentration of sulfentrazone in the soil and coefficients of variation (CV) obtained in the analyzes of the fortified Red-Yellow Oxisol samples at three levels of concentration, with three replicates at each level, for the different days of analysis.

Applied Concentration (mg L^{-1})	Concentration in the Soil (mg kg^{-1})			Mean (mg kg^{-1})	CV (%)
	Day 1	Day 2	Day 3		
			**Sulfentrazone**		
0.2	0.17	0.19	0.17	0.18	5.90
1.0	0.60	0.71	0.79	0.70	13.07
2.0	1.28	1.43	1.43	1.38	6.65
			**Sulfentrazone + Roundup Ready®**		
0.2	0.17	1.18	0.20	0.18	8.38
1.0	0.62	0.68	0.74	0.68	9.03
2.0	1.16	1.32	1.41	1.30	9.47
			**Sulfentrazone + Roundup Ultra®**		
0.2	0.18	0.20	0.20	0.19	7.43
1.0	0.64	0.74	0.74	0.71	8.02
2.0	1.09	1.43	1.44	1.32	15.00
			**Sulfentrazone + ZappQi®**		
0.2	0.18	0.20	0.22	0.20	10.14
1.0	0.63	0.74	0.76	0.71	9.21
2.0	1.18	1.40	1.47	1.35	11.58

The variation resulting from the repetition of the extraction technique on three different days can be observed in Table S4 and is expressed by the coefficient of variation. It was verified through the intermediary precision that the ESL/PBT-CLAE-UV/Vis method is suitable for sulfentrazone, since the coefficients of variation obtained are less than 20%.

The validated method is selective, precise, accurate, has good linearity within the working range and good limits of detection and quantification, proving to be adequate for the quantification of sulfentrazone in isolated application and in mixture with glyphosate formulations through the ESL/PBT-CLAE-UV/Vis.

2. Electronic Supplementary Material-2

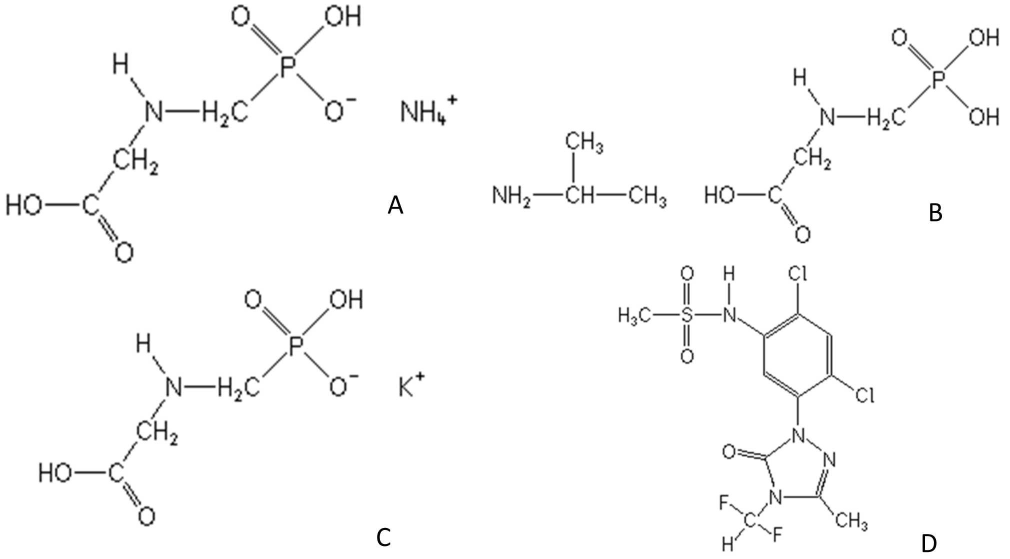

Figure S5. Molecular structures of salts of ammonium (**A**), isopropylamine (**B**) and potassium (**C**), and sulfentrazone (**D**).
